# *catena*-Poly[[[aqua­cadmium(II)]bis­(μ-4-hydroxy­pyridine-2,6-dicarboxyl­ato)[aqua­cadmium(II)]di-μ-aqua] tetra­hydrate]

**DOI:** 10.1107/S1600536808030869

**Published:** 2008-09-30

**Authors:** Hossein Aghabozorg, Neda Ilaie, Mohammad Heidari, Faranak Manteghi, Hoda Pasdar

**Affiliations:** a Faculty of Chemistry, Tarbiat Moallem University, Tehran, Iran; b Faculty of Chemistry, Islamic Azad University, North Tehran Branch, Tehran, Iran; c Faculty of Chemistry, Iran University of Science and Technology, Tehran, Iran

## Abstract

The title polymeric compound, {[Cd_2_(C_7_H_3_NO_5_)_2_(H_2_O)_4_]·4H_2_O}_*n*_ or {[Cd_2_(hypydc)_2_(H_2_O)_4_]·4H_2_O}_*n*_ (where hypydcH_2_ is 4-hydroxy­pyridine-2,6-dicarboxylic acid), was synthesized by the reaction of cadmium(II) nitrate hexa­hydrate with 4-hydroxy­pyridine-2,6-dicarboxylic acid and propane-1,3-diamine, in a 1:2:2 molar ratio in aqueous solution. The compound is a seven-coordinate binuclear polymeric complex with distorted penta­gonal bipyramidal geometry around Cd^II^ [Cd—O = 2.247 (4)–2.474 (3) Å]. In the binuclear monomeric units, the central atoms join together by O atoms of two bridging tridentate (hypydc)^2−^ ligands, and the polymer propagates *via* two bridging water mol­ecules that link each Cd^II ^centre of one monomer to the adjacent neighbour. Propane-1,3-diamine (pn) does not appear in the product but plays a role as a base. Inter­molecular O—H⋯O and C—H⋯O hydrogen bonds, and π–π stacking inter­actions, with distances of 3.725 (3) and 3.766 (3) Å, connect the various components.

## Related literature

For a review of proton-transfer compounds, see: Aghabozorg, Manteghi & Sheshmani (2008 ▶). For related compounds, see: Aghabozorg *et al.* (2007 ▶); Aghabozorg, Motyeian *et al.* (2008 ▶); Aghabozorg, Roshan *et al.* (2008 ▶); Fu *et al.* (2004 ▶); Odoko *et al.* (2002 ▶); Ranjbar *et al.* (2002 ▶); Wu *et al.* (2007 ▶). For the isostructural Mn compound, see: Ghosh *et al.* (2005 ▶).
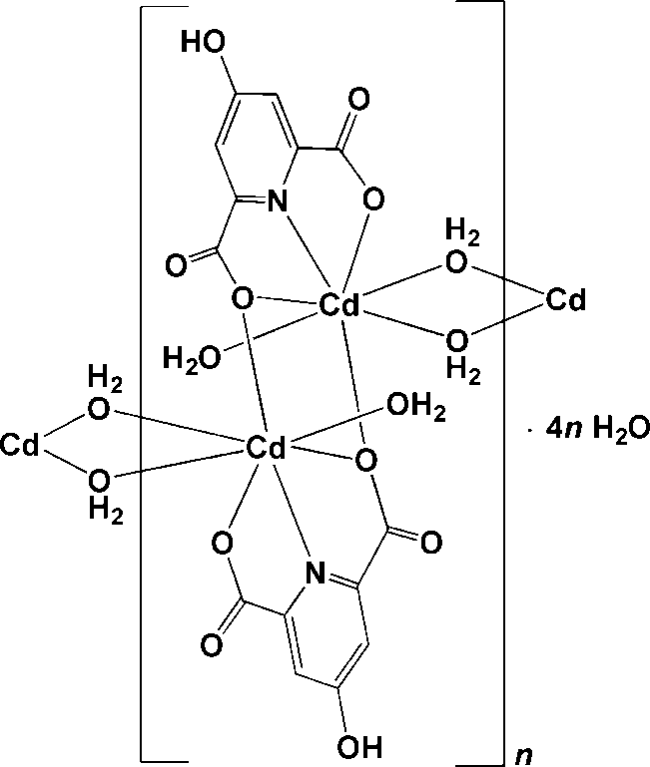


## Experimental

### 

#### Crystal data

[Cd_2_(C_7_H_3_NO_5_)_2_(H_2_O)_4_]·4H_2_O
*M* *_r_* = 731.14
Triclinic, 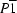

*a* = 9.4499 (6) Å
*b* = 10.8633 (7) Å
*c* = 11.2086 (9) Å
α = 87.910 (3)°
β = 74.239 (2)°
γ = 80.478 (2)°
*V* = 1092.08 (13) Å^3^
*Z* = 2
Mo *K*α radiation
μ = 2.04 mm^−1^
*T* = 100 (2) K
0.15 × 0.12 × 0.08 mm

#### Data collection

Bruker SMART APEXII diffractometer
Absorption correction: multi-scan (*SADABS*; Bruker, 2001 ▶) *T*_min_ = 0.749, *T*_max_ = 0.854
14393 measured reflections
6313 independent reflections
4460 reflections with *I* > 2σ(*I*)
*R*_int_ = 0.059

#### Refinement

*R*[*F* ^2^ > 2σ(*F* ^2^)] = 0.052
*wR*(*F* ^2^) = 0.091
*S* = 1.00
6313 reflections
265 parameters
H-atom parameters constrained
Δρ_max_ = 1.03 e Å^−3^
Δρ_min_ = −1.01 e Å^−3^

### 

Data collection: *APEX2* (Bruker, 2007 ▶); cell refinement: *SAINT* (Bruker, 2007 ▶); data reduction: *SAINT*; program(s) used to solve structure: *SHELXTL* (Sheldrick, 2008 ▶); program(s) used to refine structure: *SHELXTL*; molecular graphics: *SHELXTL*; software used to prepare material for publication: *SHELXTL*.

## Supplementary Material

Crystal structure: contains datablocks I, global. DOI: 10.1107/S1600536808030869/om2258sup1.cif

Structure factors: contains datablocks I. DOI: 10.1107/S1600536808030869/om2258Isup2.hkl

Additional supplementary materials:  crystallographic information; 3D view; checkCIF report

## Figures and Tables

**Table 1:** Hydrogen-bond geometry (Å, °)

*D*—H⋯*A*	*D*—H	H⋯*A*	*D*⋯*A*	*D*—H⋯*A*
O3—H3O⋯O9^i^	0.85	1.74	2.536 (5)	156
O6—H6*A*⋯O4^ii^	0.85	1.86	2.665 (5)	156
O6—H6*B*⋯O17	0.85	1.83	2.677 (8)	177
O7—H7*A*⋯O11^iii^	0.85	2.06	2.871 (5)	158
O7—H7*B*⋯O1^iv^	0.85	1.84	2.639 (5)	156
O8—H8*A*⋯O12^v^	0.85	1.88	2.687 (5)	159
O8—H8*B*⋯O15	0.85	1.83	2.679 (5)	176
O11—H11O⋯O5^iii^	0.85	1.76	2.547 (5)	153
O14—H14*A*⋯O18^vi^	0.85	1.94	2.747 (7)	159
O14—H14*B*⋯O16^v^	0.85	1.82	2.663 (6)	169
O15—H15*A*⋯O17^vii^	0.85	1.96	2.802 (7)	169
O15—H15*B*⋯O3^i^	0.85	2.03	2.790 (5)	149
O16—H16*A*⋯O18^viii^	0.85	2.30	3.013 (6)	142
O16—H16*A*⋯O14^ix^	0.85	2.50	3.228 (6)	143
O16—H16*B*⋯O15^ix^	0.85	1.97	2.791 (6)	161
O18—H18*A*⋯O9^ii^	0.85	1.88	2.719 (5)	170
O18—H18*B*⋯O12^x^	0.85	2.20	2.819 (6)	129
C11—H11*A*⋯O4^iii^	0.95	2.31	3.224 (6)	161

Symmetry codes: (i) 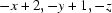; (ii) 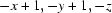; (iii) 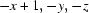; (iv) 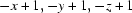; (v) 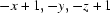; (vi) 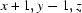; (vii) 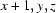; (viii) 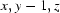; (ix) 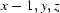; (x) 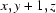.

**Fig. 1.**
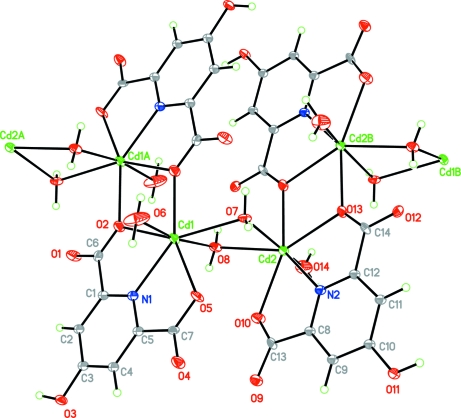
The molecular structure of the title compound as a fragment of the polymeric chain. Displacement ellipsoids are drawn at 50% probability level. Symmetry codes to generate atoms with labels: A: -x + 1,-y + 1,-z + 1; B: -x + 1, -y, -z + 1.

**Fig. 2.**
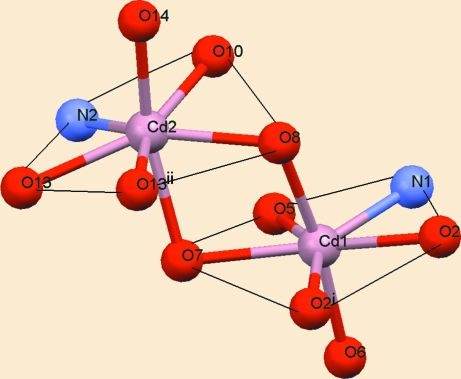
Coordination polyhedron of the title compound [i: (1 - x, 1 - y, 1 - z) and ii: (1 - x, -y, 1 - z)]

**Fig. 3.**
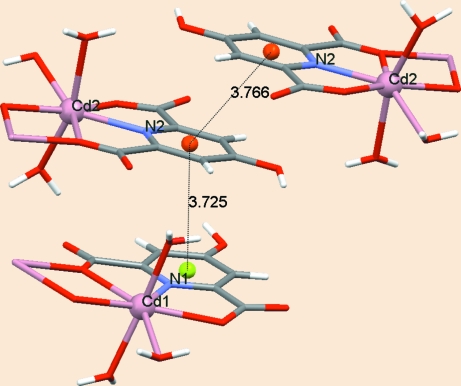
π–π Stacking interactions of Cg1–Cg2 (Cg1: N1/C1–C5; Cg2: N2/C8–C12) and Cg2–Cg2 of the title compound. The average distances between the planes are 3.725 (3) Å (x, 1 + y, z) and 3.766 (3) Å (1 - x, -y, -z), respectively.

**Fig. 4.**
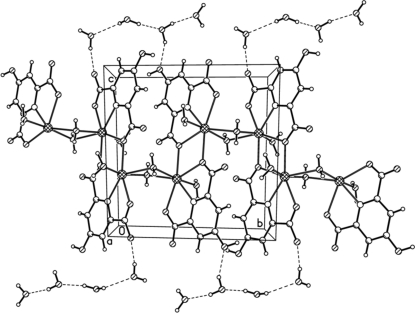
The crystal packing of the title compound along a crystal axis. Hydrogen bonds are shown as dashed lines.

**Fig. 5.**
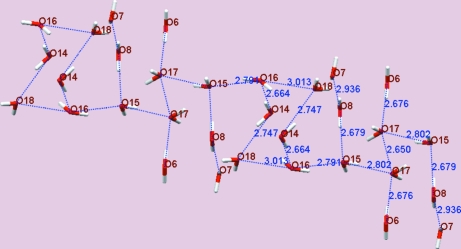
A perspective view of the water cluster arranged in the structure.

## supplementary crystallographic information

### Comment

We have reported a number of cases in which a proton is transferred from a carboxylic acid to an amine to form some novel organic compounds. This work was recently reviewed (Aghabozorg, Manteghi & Sheshmani, 2008). With the use of these organic proton transfer compounds as starting materials, several metal organic compounds were prepared. Recently, we have combined the acid, amine and metallic salt in a one-pot reaction, including the title compound in this article, [Cd_2_(hypydc)_2_(H_2_O)_4_]}*_n_*.4*n*H_2_O (where hypydcH_2_ is 4-hydroxypyridine-2,6-dicarboxylic acid). A search of the literature shows that there are similar compounds to the title compound using pydc (pydcH_2_ = pyridine-2,6-dicarboxylic acid) as ligand to Cd^II^ such as (enH_2_)_2_[Cd(pydc)_3_].6H_2_O, 1 (Fu *et al.*, 2004), or [Cd_2_(pydc)_2_(H_2_O)_6_].2pydcH_2_, 2(Odoko *et al.*, 2002), [Cd_2_(pydc)_2_(CH_3_OH)_2_(H_2_O)]*_n_*, 3 (Wu *et al.*, 2007), [Cd(pydc)(H_2_O)_3_]_2_.2H_2_pydc, 4 (Ranjbar *et al.*, 2002), [Cd(py-2,3-dc)(H_2_O)_3_]*_n_*, 5, py-2,3-dc is pyridine-2,3-dicarboxylate, (Aghabozorg, Motyeian *et al.*, 2008) or hypydc as ligand to different metals such as (pydaH)[Cr(hypydc)_2_].2H_2_O (Aghabozorg, Roshan, *et al.*, 2008), [Ni(hypydc)(H_2_O)_3_].1.5H_2_O (Aghabozorg, Ghadermazi, *et al.*, 2007).

The molecular structure, coordination polyhedra, π–π stacking, packing diagram and water cluster of the title compound are shown in Figs. 1–5. The extensive hydrogen bonding geometry is given in Table 1. Each of the two Cd^II^ atoms in the asymmetric unit is seven-coordinate. The coordination environment is distorted pentagonal bipyramidal, with two O and one N atoms of the (hypydc)^2-^ group as well as one O atom of an inversion-related (hypydc)^2-^ group and a bridging water O atom forming the pentagonal plane. The other bridging water O and one terminal O atom of a coordinated water form the apical groups (Figs. 1 and 2). The sums of the bond angles in the pentagonal plane around Cd1 and Cd2 are 362.05° and 362.89°, respectively. As shown in Fig. 1, the Cd1 and Cd2 atoms join together by O atoms of two bridging water molecules (O7 and O8), and the O atoms of tridentate (hypydc)^2-^ ligands (O2 and O13) bridge Cd1 to Cd1A and Cd2 to Cd2B to make a polymeric feature. The compound is isostructural to {[Mn_2_(hypydc)_2_(H_2_O)_4_]}*_n_*.4*n*H_2_O which has been described as propagating in a one-dimensional staircase model (Ghosh *et al.*, 2005).

Compared with the similar structures mentioned above and listed in Table 2, with various coordination numbers of six, seven and nine, the Cd—O distances of the title compound (average 2.388 Å) lie in the same range as compounds 2, 3, and 4 and far from compounds 1 and 5. However, the Cd—N distances (average 2.273 Å) are obviously shorter than all five compounds. Moreover, in the polymeric compound 3, the binuclear units are connected *via* carboxylate O atoms to build a one-dimensional polymeric chain, and in 5, the chain propagates *via* linking two oxygen atoms of (py-2,3-dc)^2-^ to cadmium centers, while in the title compound, the bridging water molecules cause propagation of the binuclear unit.

An outstanding feature of the title compound is the presence of π–π stacking interactions between aromatic rings, *Cg*1–*Cg*2 (*Cg*1: N1/C1–C5; *Cg*2: N2/C8–C12) with distances of 3.725 (3) Å (*x*, 1 + *y*, *z*) and *Cg*2–*Cg*2 with distances of 3.767 (3) Å (1 - *x*, -*y*, -*z*), as shown in Fig. 3. Intermolecular O—H···O and C—H···O hydrogen bonds with D···A ranging from 2.534 (5) Å to 3.225 (6) Å (Table 1), ion pairing and π–π stacking interactions are observed. The arrangement of water molecules in the structure consists of an R6 motif coupled to a branched C10 motif as shown in Fig. 5.

### Experimental

A solution of Cd(NO_3_)_2_.6H_2_O (172 mg, 0.5 mmol) in water (10 ml) was added to an aqueous solution of propane-1,3-diamine(74 mg, 1 mmol) and 4-hydroxypyridine-2,6-dicarboxylic acid (167 mg, 1 mmol) in water (10 ml) in a 1:2:2 molar ratio and heated for two hours. Colourless crystals of the title compound were obtained after allowing the mixture to stand for four months at room temperature.

### Refinement

The H atoms of the OH-groups and the water molecules were located in the difference Fourier map and all O—H distancies were normalized at 0.85 Å. The H(O) atoms were refined in rigid model with fixed thermal (*U*_iso_(H) = 1.2Ueq(O)) parameters. The H(C) atoms were placed in calculated positions with r(C—H) = 0.95 Å and refined in riding model with fixed thermal parameters (*U*_iso_(H) = 1.2Ueq(C)). The *U*_eq_(O or C) are the equivalent thermal parameters of the oxygen and carbon atoms, respectively, to which corresponding H atoms are bonded.

There is a high positive residual density of 1.03 e Å^-3^ near the Cd2 center (0.77 Å) due to considerable absorption effects which could not be completely corrected.

### Crystal data

**Table:**

[Cd_2_(C_7_H_3_NO_5_)_2_(H_2_O)_4_]·4H_2_O	*Z* = 2
*M**_r_* = 731.14	*F*(000) = 720
Triclinic, *P*1	*D*_x_ = 2.223 Mg m^−^^3^
Hall symbol: -P 1	Mo *K*α radiation, λ = 0.71073 Å
*a* = 9.4499 (6) Å	Cell parameters from 1712 reflections
*b* = 10.8633 (7) Å	θ = 2.3–26.6°
*c* = 11.2086 (9) Å	µ = 2.04 mm^−^^1^
α = 87.910 (3)°	*T* = 100 K
β = 74.239 (2)°	Prism, colourless
γ = 80.478 (2)°	0.15 × 0.12 × 0.08 mm
*V* = 1092.08 (13) Å^3^	

### Data collection

**Table:**

Bruker SMART APEXII diffractometer	6313 independent reflections
Radiation source: fine-focus sealed tube	4460 reflections with *I* > 2σ(*I*)
graphite	*R*_int_ = 0.060
φ and ω scans	θ_max_ = 30.1°, θ_min_ = 1.9°
Absorption correction: multi-scan (SADABS; Bruker, 2001)	*h* = −13→13
*T*_min_ = 0.749, *T*_max_ = 0.854	*k* = −15→15
14393 measured reflections	*l* = −15→15

### Refinement

**Table:**

Refinement on *F*^2^	Primary atom site location: structure-invariant direct methods
Least-squares matrix: full	Secondary atom site location: difference Fourier map
*R*[*F*^2^ > 2σ(*F*^2^)] = 0.052	Hydrogen site location: mixed
*wR*(*F*^2^) = 0.091	H-atom parameters constrained
*S* = 1.00	*w* = 1/[σ^2^(*F*_o_^2^) + (0.020*P*)^2^ + 3.*P*] where *P* = (*F*_o_^2^ + 2*F*_c_^2^)/3
6313 reflections	(Δ/σ)_max_ < 0.001
265 parameters	Δρ_max_ = 1.03 e Å^−^^3^
0 restraints	Δρ_min_ = −1.01 e Å^−^^3^

### Special details

**Table:**

Geometry. All e.s.d.'s (except the e.s.d. in the dihedral angle between two l.s. planes) are estimated using the full covariance matrix. The cell e.s.d.'s are taken into account individually in the estimation of e.s.d.'s in distances, angles and torsion angles; correlations between e.s.d.'s in cell parameters are only used when they are defined by crystal symmetry. An approximate (isotropic) treatment of cell e.s.d.'s is used for estimating e.s.d.'s involving l.s. planes.
Refinement. Refinement of *F*^2^ against ALL reflections. The weighted *R*-factor *wR* and goodness of fit *S* are based on *F*^2^, conventional *R*-factors *R* are based on *F*, with *F* set to zero for negative *F*^2^. The threshold expression of *F*^2^ > σ(*F*^2^) is used only for calculating *R*-factors(gt) *etc*. and is not relevant to the choice of reflections for refinement. *R*-factors based on *F*^2^ are statistically about twice as large as those based on *F*, and *R*- factors based on ALL data will be even larger.

### Fractional atomic coordinates and isotropic or equivalent isotropic displacement parameters (Å^2^)

**Table:**

	*x*	*y*	*z*	*U*_iso_*/*U*_eq_	
Cd1	0.52233 (4)	0.41267 (3)	0.34288 (3)	0.01222 (9)	
Cd2	0.64542 (4)	0.07117 (3)	0.36831 (3)	0.01104 (9)	
O1	0.7671 (4)	0.7051 (4)	0.4236 (3)	0.0213 (9)	
O2	0.6031 (4)	0.5739 (3)	0.4480 (3)	0.0148 (8)	
O3	0.9775 (4)	0.7024 (3)	−0.0481 (3)	0.0161 (8)	
H3O	0.9961	0.7687	−0.0219	0.019*	
O4	0.6494 (4)	0.3865 (3)	−0.0651 (3)	0.0176 (8)	
O5	0.5617 (4)	0.3419 (3)	0.1337 (3)	0.0168 (8)	
O6	0.3269 (5)	0.5374 (4)	0.2974 (4)	0.0362 (12)	
H6A	0.3554	0.5447	0.2191	0.043*	
H6B	0.2343	0.5340	0.3225	0.043*	
O7	0.4213 (4)	0.2185 (3)	0.3620 (3)	0.0125 (7)	
H7A	0.3838	0.2156	0.3015	0.015*	
H7B	0.3509	0.2240	0.4284	0.015*	
O8	0.6944 (4)	0.2700 (3)	0.4137 (3)	0.0131 (7)	
H8A	0.6943	0.2618	0.4894	0.016*	
H8B	0.7838	0.2795	0.3789	0.016*	
O9	0.8938 (4)	0.1332 (3)	−0.0142 (3)	0.0154 (7)	
O10	0.8252 (4)	0.1290 (4)	0.1920 (3)	0.0189 (8)	
O11	0.6211 (4)	−0.1986 (3)	−0.1198 (3)	0.0154 (8)	
H11O	0.5712	−0.2583	−0.1067	0.018*	
O12	0.3580 (4)	−0.2089 (3)	0.3456 (3)	0.0146 (7)	
O13	0.4717 (4)	−0.0785 (3)	0.4216 (3)	0.0141 (7)	
O14	0.8487 (4)	−0.0278 (4)	0.4192 (4)	0.0261 (9)	
H14A	0.9137	−0.0794	0.3697	0.031*	
H14B	0.8540	−0.0580	0.4892	0.031*	
N1	0.6853 (5)	0.5189 (4)	0.2066 (4)	0.0119 (4)	
N2	0.6319 (4)	−0.0249 (4)	0.1969 (4)	0.0104 (4)	
C1	0.7505 (5)	0.6044 (5)	0.2457 (4)	0.0119 (4)	
C2	0.8515 (5)	0.6687 (4)	0.1641 (4)	0.0119 (4)	
H2A	0.8979	0.7272	0.1945	0.014*	
C3	0.8838 (5)	0.6462 (5)	0.0370 (4)	0.0119 (4)	
C4	0.8116 (5)	0.5594 (4)	−0.0030 (4)	0.0119 (4)	
H4A	0.8286	0.5436	−0.0890	0.014*	
C5	0.7155 (5)	0.4969 (4)	0.0840 (4)	0.0119 (4)	
C6	0.7047 (6)	0.6299 (5)	0.3840 (5)	0.0149 (10)	
C7	0.6364 (5)	0.4018 (5)	0.0464 (4)	0.0107 (9)	
C8	0.7203 (5)	−0.0014 (4)	0.0849 (4)	0.0104 (4)	
C9	0.7187 (5)	−0.0582 (4)	−0.0220 (4)	0.0104 (4)	
H9A	0.7824	−0.0395	−0.0996	0.012*	
C10	0.6223 (5)	−0.1432 (4)	−0.0149 (4)	0.0104 (4)	
C11	0.5281 (5)	−0.1673 (4)	0.1005 (4)	0.0104 (4)	
H11A	0.4595	−0.2238	0.1076	0.012*	
C12	0.5376 (5)	−0.1068 (4)	0.2040 (4)	0.0104 (4)	
C13	0.8203 (6)	0.0931 (5)	0.0898 (5)	0.0121 (10)	
C14	0.4476 (6)	−0.1331 (5)	0.3343 (4)	0.0113 (10)	
O15	0.9715 (4)	0.3122 (4)	0.3047 (3)	0.0203 (8)	
H15A	0.9866	0.3760	0.3381	0.024*	
H15B	0.9582	0.3302	0.2337	0.024*	
O16	0.1500 (5)	0.0936 (4)	0.3498 (4)	0.0355 (11)	
H16A	0.0945	0.0451	0.3354	0.043*	
H16B	0.1150	0.1647	0.3267	0.043*	
O17	0.0340 (6)	0.5346 (5)	0.3824 (6)	0.0617 (17)	
H17A	−0.0011	0.5732	0.3266	0.074*	
H17B	0.0098	0.5884	0.4407	0.074*	
O18	0.1026 (5)	0.8506 (6)	0.2576 (4)	0.0601 (18)	
H18A	0.1070	0.8458	0.1811	0.072*	
H18B	0.1561	0.7904	0.2832	0.072*	

### Atomic displacement parameters (Å^2^)

**Table:**

	*U*^11^	*U*^22^	*U*^33^	*U*^12^	*U*^13^	*U*^23^
Cd1	0.0173 (2)	0.0132 (2)	0.00806 (18)	−0.00777 (16)	−0.00365 (15)	0.00109 (14)
Cd2	0.01285 (19)	0.01276 (19)	0.00844 (18)	−0.00532 (15)	−0.00244 (15)	−0.00044 (14)
O1	0.031 (2)	0.028 (2)	0.0082 (17)	−0.0200 (18)	−0.0008 (16)	−0.0055 (15)
O2	0.0185 (19)	0.0151 (19)	0.0115 (18)	−0.0074 (15)	−0.0024 (15)	−0.0002 (14)
O3	0.0175 (19)	0.0163 (19)	0.0130 (18)	−0.0089 (15)	0.0024 (15)	−0.0004 (14)
O4	0.027 (2)	0.019 (2)	0.0095 (17)	−0.0118 (16)	−0.0043 (15)	0.0014 (14)
O5	0.026 (2)	0.0194 (19)	0.0077 (17)	−0.0168 (16)	−0.0011 (15)	0.0009 (14)
O6	0.025 (2)	0.063 (3)	0.013 (2)	0.016 (2)	−0.0060 (18)	−0.002 (2)
O7	0.0106 (17)	0.0196 (19)	0.0072 (16)	−0.0046 (14)	−0.0003 (13)	−0.0022 (13)
O8	0.0149 (18)	0.0188 (19)	0.0068 (16)	−0.0053 (14)	−0.0036 (14)	0.0015 (13)
O9	0.0179 (19)	0.0188 (19)	0.0115 (17)	−0.0097 (15)	−0.0034 (14)	0.0001 (14)
O10	0.025 (2)	0.025 (2)	0.0087 (17)	−0.0142 (17)	−0.0024 (15)	−0.0027 (15)
O11	0.022 (2)	0.0192 (19)	0.0074 (16)	−0.0142 (15)	−0.0021 (14)	−0.0031 (14)
O12	0.022 (2)	0.0134 (18)	0.0091 (17)	−0.0050 (15)	−0.0028 (15)	−0.0040 (13)
O13	0.0194 (19)	0.0139 (18)	0.0114 (17)	−0.0098 (15)	−0.0038 (15)	−0.0016 (14)
O14	0.027 (2)	0.032 (2)	0.018 (2)	−0.0006 (19)	−0.0072 (18)	0.0077 (17)
N1	0.0120 (10)	0.0121 (10)	0.0104 (9)	−0.0027 (7)	−0.0003 (7)	−0.0008 (7)
N2	0.0103 (9)	0.0122 (10)	0.0084 (9)	−0.0023 (7)	−0.0017 (7)	0.0000 (7)
C1	0.0120 (10)	0.0121 (10)	0.0104 (9)	−0.0027 (7)	−0.0003 (7)	−0.0008 (7)
C2	0.0120 (10)	0.0121 (10)	0.0104 (9)	−0.0027 (7)	−0.0003 (7)	−0.0008 (7)
C3	0.0120 (10)	0.0121 (10)	0.0104 (9)	−0.0027 (7)	−0.0003 (7)	−0.0008 (7)
C4	0.0120 (10)	0.0121 (10)	0.0104 (9)	−0.0027 (7)	−0.0003 (7)	−0.0008 (7)
C5	0.0120 (10)	0.0121 (10)	0.0104 (9)	−0.0027 (7)	−0.0003 (7)	−0.0008 (7)
C6	0.016 (3)	0.010 (2)	0.017 (3)	−0.004 (2)	0.000 (2)	−0.0035 (19)
C7	0.007 (2)	0.014 (2)	0.010 (2)	−0.0031 (18)	0.0009 (18)	−0.0028 (18)
C8	0.0103 (9)	0.0122 (10)	0.0084 (9)	−0.0023 (7)	−0.0017 (7)	0.0000 (7)
C9	0.0103 (9)	0.0122 (10)	0.0084 (9)	−0.0023 (7)	−0.0017 (7)	0.0000 (7)
C10	0.0103 (9)	0.0122 (10)	0.0084 (9)	−0.0023 (7)	−0.0017 (7)	0.0000 (7)
C11	0.0103 (9)	0.0122 (10)	0.0084 (9)	−0.0023 (7)	−0.0017 (7)	0.0000 (7)
C12	0.0103 (9)	0.0122 (10)	0.0084 (9)	−0.0023 (7)	−0.0017 (7)	0.0000 (7)
C13	0.013 (2)	0.013 (2)	0.014 (2)	−0.0096 (19)	−0.0051 (19)	0.0017 (18)
C14	0.013 (2)	0.011 (2)	0.011 (2)	−0.0052 (19)	−0.0050 (19)	0.0063 (18)
O15	0.0175 (19)	0.033 (2)	0.0114 (18)	−0.0112 (17)	−0.0013 (15)	−0.0039 (16)
O16	0.052 (3)	0.037 (3)	0.018 (2)	−0.012 (2)	−0.009 (2)	0.0051 (19)
O17	0.036 (3)	0.055 (4)	0.098 (5)	0.003 (3)	−0.026 (3)	−0.040 (3)
O18	0.031 (3)	0.127 (5)	0.012 (2)	0.029 (3)	−0.011 (2)	−0.013 (3)

### Geometric parameters (Å, °)

**Table:**

Cd1—O6	2.267 (4)	O13—C14	1.252 (6)
Cd1—N1	2.284 (4)	O13—Cd2^ii^	2.312 (3)
Cd1—O2^i^	2.320 (3)	O14—H14A	0.8500
Cd1—O8	2.335 (3)	O14—H14B	0.8500
Cd1—O5	2.406 (3)	N1—C1	1.343 (6)
Cd1—O7	2.435 (3)	N1—C5	1.347 (6)
Cd1—O2	2.474 (3)	N2—C12	1.346 (6)
Cd2—O14	2.244 (4)	N2—C8	1.347 (6)
Cd2—N2	2.263 (4)	C1—C2	1.390 (7)
Cd2—O13^ii^	2.312 (3)	C1—C6	1.515 (7)
Cd2—O10	2.372 (4)	C2—C3	1.395 (7)
Cd2—O8	2.383 (3)	C2—H2A	0.9500
Cd2—O13	2.445 (3)	C3—C4	1.402 (7)
Cd2—O7	2.450 (3)	C4—C5	1.383 (7)
O1—C6	1.241 (6)	C4—H4A	0.9500
O2—C6	1.262 (6)	C5—C7	1.505 (7)
O2—Cd1^i^	2.320 (3)	C8—C9	1.373 (6)
O3—C3	1.320 (5)	C8—C13	1.518 (6)
O3—H3O	0.8500	C9—C10	1.387 (6)
O4—C7	1.237 (6)	C9—H9A	0.9500
O5—C7	1.269 (6)	C10—C11	1.400 (6)
O6—H6A	0.8500	C11—C12	1.385 (6)
O6—H6B	0.8500	C11—H11A	0.9500
O7—H7A	0.8501	C12—C14	1.521 (7)
O7—H7B	0.8500	O15—H15A	0.8500
O8—H8A	0.8500	O15—H15B	0.8500
O8—H8B	0.8500	O16—H16A	0.8500
O9—C13	1.286 (6)	O16—H16B	0.8500
O10—C13	1.238 (6)	O17—H17A	0.8499
O11—C10	1.344 (5)	O17—H17B	0.8501
O11—H11O	0.8500	O18—H18A	0.8500
O12—C14	1.255 (6)	O18—H18B	0.8500
			
O6—Cd1—N1	90.59 (16)	H8A—O8—H8B	102.3
O6—Cd1—O2^i^	90.18 (14)	C13—O10—Cd2	116.8 (3)
N1—Cd1—O2^i^	136.84 (13)	C10—O11—H11O	113.0
O6—Cd1—O8	170.72 (15)	C14—O13—Cd2^ii^	131.0 (3)
N1—Cd1—O8	98.66 (13)	C14—O13—Cd2	117.7 (3)
O2^i^—Cd1—O8	82.57 (12)	Cd2^ii^—O13—Cd2	110.75 (13)
O6—Cd1—O5	81.50 (14)	Cd2—O14—H14A	121.4
N1—Cd1—O5	69.20 (13)	Cd2—O14—H14B	127.6
O2^i^—Cd1—O5	153.07 (12)	H14A—O14—H14B	101.5
O8—Cd1—O5	102.43 (12)	C1—N1—C5	118.9 (4)
O6—Cd1—O7	97.28 (15)	C1—N1—Cd1	121.5 (3)
N1—Cd1—O7	141.26 (13)	C5—N1—Cd1	119.6 (3)
O2^i^—Cd1—O7	81.24 (11)	C12—N2—C8	118.6 (4)
O8—Cd1—O7	75.95 (12)	C12—N2—Cd2	121.3 (3)
O5—Cd1—O7	74.55 (11)	C8—N2—Cd2	120.1 (3)
O6—Cd1—O2	96.88 (15)	N1—C1—C2	122.2 (4)
N1—Cd1—O2	67.99 (13)	N1—C1—C6	116.1 (4)
O2^i^—Cd1—O2	69.08 (14)	C2—C1—C6	121.6 (4)
O8—Cd1—O2	85.97 (12)	C1—C2—C3	119.2 (5)
O5—Cd1—O2	137.14 (11)	C1—C2—H2A	120.4
O7—Cd1—O2	147.01 (11)	C3—C2—H2A	120.4
O14—Cd2—N2	107.13 (15)	O3—C3—C2	124.0 (4)
O14—Cd2—O13^ii^	86.47 (13)	O3—C3—C4	117.9 (4)
N2—Cd2—O13^ii^	137.82 (13)	C2—C3—C4	118.1 (4)
O14—Cd2—O10	82.66 (14)	C5—C4—C3	119.3 (4)
N2—Cd2—O10	70.05 (13)	C5—C4—H4A	120.4
O13^ii^—Cd2—O10	152.13 (12)	C3—C4—H4A	120.4
O14—Cd2—O8	91.90 (13)	N1—C5—C4	122.2 (5)
N2—Cd2—O8	135.50 (13)	N1—C5—C7	116.2 (4)
O13^ii^—Cd2—O8	81.67 (12)	C4—C5—C7	121.5 (4)
O10—Cd2—O8	73.16 (12)	O1—C6—O2	126.1 (5)
O14—Cd2—O13	103.58 (13)	O1—C6—C1	117.7 (4)
N2—Cd2—O13	68.77 (13)	O2—C6—C1	116.2 (4)
O13^ii^—Cd2—O13	69.25 (13)	O4—C7—O5	125.0 (5)
O10—Cd2—O13	138.33 (12)	O4—C7—C5	118.8 (4)
O8—Cd2—O13	145.76 (12)	O5—C7—C5	116.2 (4)
O14—Cd2—O7	163.49 (13)	N2—C8—C9	122.7 (4)
N2—Cd2—O7	89.32 (13)	N2—C8—C13	113.3 (4)
O13^ii^—Cd2—O7	82.00 (12)	C9—C8—C13	124.1 (4)
O10—Cd2—O7	102.26 (12)	C8—C9—C10	118.8 (4)
O8—Cd2—O7	74.81 (11)	C8—C9—H9A	120.6
O13—Cd2—O7	83.36 (12)	C10—C9—H9A	120.6
C6—O2—Cd1^i^	131.4 (3)	O11—C10—C9	118.6 (4)
C6—O2—Cd1	117.6 (3)	O11—C10—C11	122.2 (4)
Cd1^i^—O2—Cd1	110.92 (14)	C9—C10—C11	119.2 (4)
C3—O3—H3O	113.4	C12—C11—C10	118.3 (4)
C7—O5—Cd1	118.0 (3)	C12—C11—H11A	120.9
Cd1—O6—H6A	103.6	C10—C11—H11A	120.9
Cd1—O6—H6B	129.8	N2—C12—C11	122.3 (4)
H6A—O6—H6B	111.7	N2—C12—C14	115.4 (4)
Cd1—O7—Cd2	99.37 (12)	C11—C12—C14	122.2 (4)
Cd1—O7—H7A	106.8	O10—C13—O9	123.6 (4)
Cd2—O7—H7A	119.4	O10—C13—C8	119.1 (4)
Cd1—O7—H7B	107.0	O9—C13—C8	117.3 (4)
Cd2—O7—H7B	115.1	O13—C14—O12	125.6 (5)
H7A—O7—H7B	107.9	O13—C14—C12	116.7 (4)
Cd1—O8—Cd2	104.26 (13)	O12—C14—C12	117.6 (4)
Cd1—O8—H8A	124.0	H15A—O15—H15B	110.3
Cd2—O8—H8A	101.5	H16A—O16—H16B	104.2
Cd1—O8—H8B	112.2	H17A—O17—H17B	102.7
Cd2—O8—H8B	112.2	H18A—O18—H18B	114.0
			
O6—Cd1—O2—C6	−94.9 (4)	O13^ii^—Cd2—N2—C12	−2.7 (5)
N1—Cd1—O2—C6	−7.1 (4)	O10—Cd2—N2—C12	176.7 (4)
O2^i^—Cd1—O2—C6	177.6 (5)	O8—Cd2—N2—C12	−147.3 (3)
O8—Cd1—O2—C6	94.0 (4)	O13—Cd2—N2—C12	3.2 (3)
O5—Cd1—O2—C6	−10.0 (5)	O7—Cd2—N2—C12	−79.9 (4)
O7—Cd1—O2—C6	150.2 (3)	O14—Cd2—N2—C8	−78.0 (4)
O6—Cd1—O2—Cd1^i^	87.55 (17)	O13^ii^—Cd2—N2—C8	177.9 (3)
N1—Cd1—O2—Cd1^i^	175.4 (2)	O10—Cd2—N2—C8	−2.7 (3)
O2^i^—Cd1—O2—Cd1^i^	0.0	O8—Cd2—N2—C8	33.3 (4)
O8—Cd1—O2—Cd1^i^	−83.58 (15)	O13—Cd2—N2—C8	−176.2 (4)
O5—Cd1—O2—Cd1^i^	172.46 (14)	O7—Cd2—N2—C8	100.6 (4)
O7—Cd1—O2—Cd1^i^	−27.3 (3)	C5—N1—C1—C2	−1.8 (7)
O6—Cd1—O5—C7	86.1 (4)	Cd1—N1—C1—C2	179.1 (4)
N1—Cd1—O5—C7	−7.8 (3)	C5—N1—C1—C6	176.5 (4)
O2^i^—Cd1—O5—C7	159.4 (3)	Cd1—N1—C1—C6	−2.5 (6)
O8—Cd1—O5—C7	−102.5 (4)	N1—C1—C2—C3	1.6 (8)
O7—Cd1—O5—C7	−173.9 (4)	C6—C1—C2—C3	−176.6 (5)
O2—Cd1—O5—C7	−4.9 (5)	C1—C2—C3—O3	179.8 (4)
O6—Cd1—O7—Cd2	168.82 (13)	C1—C2—C3—C4	0.3 (7)
N1—Cd1—O7—Cd2	68.7 (2)	O3—C3—C4—C5	178.5 (4)
O2^i^—Cd1—O7—Cd2	−102.13 (13)	C2—C3—C4—C5	−1.9 (7)
O8—Cd1—O7—Cd2	−17.66 (11)	C1—N1—C5—C4	0.1 (7)
O5—Cd1—O7—Cd2	89.76 (13)	Cd1—N1—C5—C4	179.2 (4)
O2—Cd1—O7—Cd2	−76.4 (2)	C1—N1—C5—C7	−178.9 (4)
O14—Cd2—O7—Cd1	54.7 (5)	Cd1—N1—C5—C7	0.2 (6)
N2—Cd2—O7—Cd1	−120.54 (14)	C3—C4—C5—N1	1.8 (8)
O13^ii^—Cd2—O7—Cd1	100.87 (13)	C3—C4—C5—C7	−179.3 (4)
O10—Cd2—O7—Cd1	−51.13 (13)	Cd1^i^—O2—C6—O1	3.4 (8)
O8—Cd2—O7—Cd1	17.39 (11)	Cd1—O2—C6—O1	−173.5 (4)
O13—Cd2—O7—Cd1	170.76 (12)	Cd1^i^—O2—C6—C1	−174.8 (3)
N1—Cd1—O8—Cd2	−122.31 (14)	Cd1—O2—C6—C1	8.2 (6)
O2^i^—Cd1—O8—Cd2	101.29 (14)	N1—C1—C6—O1	177.4 (5)
O5—Cd1—O8—Cd2	−51.83 (14)	C2—C1—C6—O1	−4.2 (8)
O7—Cd1—O8—Cd2	18.52 (11)	N1—C1—C6—O2	−4.2 (7)
O2—Cd1—O8—Cd2	170.69 (14)	C2—C1—C6—O2	174.2 (5)
O14—Cd2—O8—Cd1	171.44 (14)	Cd1—O5—C7—O4	−170.3 (4)
N2—Cd2—O8—Cd1	54.4 (2)	Cd1—O5—C7—C5	10.5 (6)
O13^ii^—Cd2—O8—Cd1	−102.41 (14)	N1—C5—C7—O4	173.4 (5)
O10—Cd2—O8—Cd1	89.69 (15)	C4—C5—C7—O4	−5.6 (7)
O13—Cd2—O8—Cd1	−70.8 (2)	N1—C5—C7—O5	−7.4 (7)
O7—Cd2—O8—Cd1	−18.49 (11)	C4—C5—C7—O5	173.7 (5)
O14—Cd2—O10—C13	118.3 (4)	C12—N2—C8—C9	−0.4 (7)
N2—Cd2—O10—C13	7.0 (4)	Cd2—N2—C8—C9	179.0 (4)
O13^ii^—Cd2—O10—C13	−173.8 (3)	C12—N2—C8—C13	179.6 (4)
O8—Cd2—O10—C13	−147.5 (4)	Cd2—N2—C8—C13	−1.0 (5)
O13—Cd2—O10—C13	16.1 (5)	N2—C8—C9—C10	0.0 (7)
O7—Cd2—O10—C13	−77.7 (4)	C13—C8—C9—C10	180.0 (4)
O14—Cd2—O13—C14	−107.3 (4)	C8—C9—C10—O11	−180.0 (4)
N2—Cd2—O13—C14	−3.9 (4)	C8—C9—C10—C11	1.0 (7)
O13^ii^—Cd2—O13—C14	171.9 (5)	O11—C10—C11—C12	179.5 (4)
O10—Cd2—O13—C14	−13.1 (5)	C9—C10—C11—C12	−1.4 (7)
O8—Cd2—O13—C14	138.2 (3)	C8—N2—C12—C11	−0.1 (7)
O7—Cd2—O13—C14	87.9 (4)	Cd2—N2—C12—C11	−179.6 (4)
O14—Cd2—O13—Cd2^ii^	80.84 (17)	C8—N2—C12—C14	177.0 (4)
N2—Cd2—O13—Cd2^ii^	−175.8 (2)	Cd2—N2—C12—C14	−2.5 (6)
O13^ii^—Cd2—O13—Cd2^ii^	0.0	C10—C11—C12—N2	1.0 (7)
O10—Cd2—O13—Cd2^ii^	175.07 (15)	C10—C11—C12—C14	−175.9 (4)
O8—Cd2—O13—Cd2^ii^	−33.7 (3)	Cd2—O10—C13—O9	168.5 (4)
O7—Cd2—O13—Cd2^ii^	−83.93 (15)	Cd2—O10—C13—C8	−10.2 (6)
O6—Cd1—N1—C1	101.9 (4)	N2—C8—C13—O10	7.7 (7)
O2^i^—Cd1—N1—C1	11.0 (5)	C9—C8—C13—O10	−172.3 (5)
O8—Cd1—N1—C1	−77.4 (4)	N2—C8—C13—O9	−171.1 (4)
O5—Cd1—N1—C1	−177.5 (4)	C9—C8—C13—O9	8.9 (7)
O7—Cd1—N1—C1	−155.7 (3)	Cd2^ii^—O13—C14—O12	−7.4 (8)
O2—Cd1—N1—C1	4.7 (4)	Cd2—O13—C14—O12	−177.3 (4)
O6—Cd1—N1—C5	−77.2 (4)	Cd2^ii^—O13—C14—C12	174.0 (3)
O2^i^—Cd1—N1—C5	−168.1 (3)	Cd2—O13—C14—C12	4.1 (6)
O8—Cd1—N1—C5	103.6 (4)	N2—C12—C14—O13	−1.3 (7)
O5—Cd1—N1—C5	3.5 (3)	C11—C12—C14—O13	175.8 (5)
O7—Cd1—N1—C5	25.2 (5)	N2—C12—C14—O12	180.0 (4)
O2—Cd1—N1—C5	−174.4 (4)	C11—C12—C14—O12	−2.9 (7)
O14—Cd2—N2—C12	101.5 (4)		

Symmetry codes: (i) −*x*+1, −*y*+1, −*z*+1; (ii) −*x*+1, −*y*, −*z*+1.

### Hydrogen-bond geometry (Å, °)

**Table:**

*D*—H···*A*	*D*—H	H···*A*	*D*···*A*	*D*—H···*A*
O3—H3O···O9^iii^	0.85	1.74	2.536 (5)	156
O6—H6A···O4^iv^	0.85	1.87	2.665 (5)	156
O6—H6B···O17	0.85	1.83	2.677 (8)	177
O7—H7A···O11^v^	0.85	2.07	2.871 (5)	158
O7—H7B···O1^i^	0.85	1.84	2.639 (5)	156
O8—H8A···O12^ii^	0.85	1.88	2.687 (5)	159
O8—H8B···O15	0.85	1.83	2.679 (5)	176
O11—H11O···O5^v^	0.85	1.76	2.547 (5)	153
O14—H14A···O18^vi^	0.85	1.94	2.747 (7)	159
O14—H14B···O16^ii^	0.85	1.82	2.663 (6)	169
O15—H15A···O17^vii^	0.85	1.96	2.802 (7)	169
O15—H15B···O3^iii^	0.85	2.03	2.790 (5)	149
O16—H16A···O18^viii^	0.85	2.30	3.013 (6)	142
O16—H16A···O14^ix^	0.85	2.51	3.228 (6)	143
O16—H16B···O15^ix^	0.85	1.97	2.791 (6)	161
O18—H18A···O9^iv^	0.85	1.88	2.719 (5)	170
O18—H18B···O12^x^	0.85	2.20	2.819 (6)	129
C11—H11A···O4^v^	0.95	2.31	3.224 (6)	161

Symmetry codes: (iii) −*x*+2, −*y*+1, −*z*; (iv) −*x*+1, −*y*+1, −*z*;  (v) −*x*+1, −*y*, −*z*; (i) −*x*+1, −*y*+1, −*z*+1; (ii) −*x*+1, −*y*, −*z*+1; (vi) *x*+1, *y*−1, *z*; (vii) *x*+1, *y*, *z*; (viii) *x*, *y*−1, *z*; (ix) *x*−1, *y*, *z*; (x) *x*, *y*+1, *z*.

### Table 2 Cd—O and Cd—N distances in comparable compounds

**Table:**

Compound		Coordination No.	Cd—O bond length (Å)	Cd—N bond length (Å)
(enH_2_)_2_[Cd(pydc)_3_]^.^6H_2_O, 1 (Fu *et al.*, 2004)		9	2.522, 2.541, 2.567	2.397, 2.419
[Cd_2_(pydc)_2_(H_2_O)_6_]^.^2pydcH_2_, 2 (Odoko *et al.*, 2002)		7	2.376, 2.396	2.478, 2.315
[Cd_2_(pydc)_2_(CH_3_OH)_2_(H_2_O)]_n_, 3 (Wu *et al.*, 2007)		7	2.326, 2.331, 2.376, 2.464	2.319
Cd(pydc)(H_2_O)_3_]_2_^.^2H_2_pydc, 4 (Ranjbar *et al.*, 2002)		7	2.367, 2.390, 2.453	2.321
[Cd(py-2,3-dc)(H_2_O)_3_]_n_, 5 (Aghabozorg, Motyeian *et al.*, 2008)		6	2.259, 2.279	2.302

Cd—O bonds of coordinated water molecules are not considered.
